# What is the impact of dynamic contrast-enhancement sequence in the Vesical Imaging, Reporting and Data System (VI-RADS)? A subgroup analysis

**DOI:** 10.1186/s40644-022-00459-1

**Published:** 2022-05-03

**Authors:** Thaisa Gvozdenovic Medina Bricio, Gabriel Lion Gouvea, Rafael Vasconcelos Barros, Fernando Chahud, Jorge Elias, Rodolfo B. Reis, Valdair F. Muglia

**Affiliations:** 1 Department of Imaging, Clinical Oncology and Hematology – Ribeirao Preto School of Medicine, University of Sao Paulo, Av Bandeirantes 3900, Campus Monte Alegre, Ribeirao Preto, São Paulo, 14049-900 Brazil; 2 Department of Pathology - Ribeirao Preto School of Medicine, University of Sao Paulo, Ribeirao Preto , Brazil; 3 Department of Surgery and Anatomy – Urology Division - Ribeirao Preto School of Medicine, University of Sao Paulo, Ribeirao Preto, Brazil

**Keywords:** Bladder cancer, MRI, Cancer staging

## Abstract

**Background:**

A scoring system focusing on the risk of muscle layer invasion by Bladder cancer (BCa) has been released, Vesical Imaging - Radiological and Data System (VI-RADS), with a growing interest in evaluating its diagnostic accuracy.

Our goal was to assess the accuracy and reproducibility of the VI-RADS score for assessment of the vesical muscular layer with (multiparametric-mp) and without (biparametric-bp) a dynamic-contrast enhancement (DCE) sequence.

**Methods:**

Retrospective study conducted from July 2018 to July 2020. All patients had suspicions of BCa and underwent Magnetic Resonance Imaging (MRI) before any intervention. MRI was interpreted by two radiologists with different levels of experience, and a VI-RADS score assigned in two different sessions (3 months apart) without and with DCE. After exclusions, 44 patients with 50 lesions were enrolled. The standard of reference was transurethral resection in 18 patients (40.9%) and cystectomy in 26 patients (59.1%).

**Results:**

Twenty-five lesions (50%) were muscle-invasive. There was no significant difference between the two groups for gender and presence of a stalk, but mean age of NMIBCa group was significantly higher (*p* = 0.01). The sizes of lesions were significantly different between groups for both readers at 2.42+/− 1.58 vs. 5.70+/− 2.67 cm for reader 1 (*p* < 0.0001) and 2.37+/− 1.50 vs. 5.44 +/− 2.90 cm for reader 2 (*p* = 0.001). The area under the curve (AUC) for muscle invasion with mpVI-RADS, considering all lesions, was 0.885 +/− 0.04 (95% CI-0.79-0.98) for reader 1 and 0.924 +/− 0.04 (0.84–0.99) for reader 2, and for bpVI-RADS was 0.879+/− 0.05 and 0.916 +/− 0.04 (0.85–0.99), respectively, both differences not statistically significant (*p* = 0.24 and 0.07, respectively). When considering only small lesions (< 3.0 cm), the accuracy for mpVI-RADS was 0.795 +/− 0.11 (0.57–1.0) for reader1, and 0.80 +/− 0.11(0.57–1.0) for reader 2, a non-significant difference (*p* = 0.56) and for bpVI-RADS was 0.747 +/− 0.12 (0.50–0.99) for reader 1 and 0.80 +/− 0.11(0.57–1.0) for reader 2, a significant difference (*p* = 0.04). The intraclass correlation coefficient for the final score was 0.81 (0.60–1.0) for mpVI-RADS and 0.85 (0.63–1.0) for bpVI-RADS.

**Conclusion:**

The VI-RADS system was accurate in demonstrating muscle-invasive BCa, for both experienced and less experienced reader, regardless of the use of a DCE sequence. However, when only small lesions were assessed the difference between the two readers was significant only for the biparametric analysis. The reproducibility was similar between multiparametric and biparametric approach.

## Introduction

The therapeutic approach for bladder cancer (BCa) is largely driven by the clinical staging at the time of diagnosis [1, 2]. Urothelial lesions are the dominant histologic type of BCa, representing about 90% of all malignant vesical lesions. Their local staging is quite variable, and a significant proportion of the cancers is limited to the mucosa [2]. Defining the status of the muscular layer is essential to determine the treatment. Non-muscular invasive bladder cancer (NMIBC) lesions are treated conservatively, and the bladder and micturition mechanism are preserved [3, 4]. However, for muscular invasive (MIBC) lesions, the usual approach is radical cystectomy or cystoprostatectomy, depending on the extension of the primary lesion [5].

The standard way to define the extension of vesical cancers is cystoscopy followed by biopsy using transurethral procedures. Transurethral resection of bladder tumors (TURBT) can be performed as the first treatment option for most NMIBCs but can also be carried out as a diagnostic procedure for MIBCs [5]. The goal is to obtain samples from the underlying bladder wall, including the mucosa, lamina, and muscularis propria. However, the accuracy of the approach of cystoscopy plus biopsy is suboptimal, and understaging is estimated to occur in about 25% of cases [6–8].

The imaging approach for local staging of BCa had been restricted because both Computed Tomography (CT) and Magnetic Resonance Imaging (MRI) show limited accuracy [9]. But recently, several studies have indicated very promising results for defining the status of muscular layer in BCa using multiparametric MRI, a combination of T2, diffusion-weighted imaging (DWI), and dynamic contrast-enhancement (DCE). The accuracy has ranged from 92 to 94% in recent studies using a multiparametric approach [10, 11].

Based on these promising results, in 2018, a group of experts developed a scoring system to assess the status of the muscular layer in BCa [12]. The Vesical Imaging Radiological and Data System (VI-RADS) was inspired by similar tools for the standardization imaging approaches, such as BI-RADS and PI-RADS [13, 14]. After its release in 2018, some studies have performed external validations with very encouraging results, and the overall accuracy has ranged from 0.79 to 0.94 [15, 16]. Also, the level of agreement for the categorization by each single sequence and for the final category of VI-RADS has ranged from 0.82 to 0.95, and there has been almost perfect agreement in the majority of the studies so far [17, 18].

Notwithstanding these promising results, some studies have questioned the use of contrast-enhancement sequences, arguing that biparametric MRI would lead to similar results to the multiparametric approach [19, 20]. Therefore, we carried out this retrospective, single-institution study with two purposes. First, to assess if a “biparametric” VI-RADS would have similar results to the original for distinguishing NMIBC from MIBC, and, second, to perform a subgroup analysis according to tumor burden.

## Material and methods

### Patient selection

We retrospectively searched the Radiological Information System (RIS) and Hospital Information System (HIS) of our institution. We retrieved information on patients with confirmed BCa who underwent MRI from July 2018 to July 2020 and had histopathological results to evaluate the status of vesical muscular layer. We limited the search to this period to ensure that the MRI protocol suggested by the VI-RADS group was used. The Institutional Review Board approved this study with a waiver for informed consent (CAAE 29175020.0.0000.5440).

**Fig. 1:**
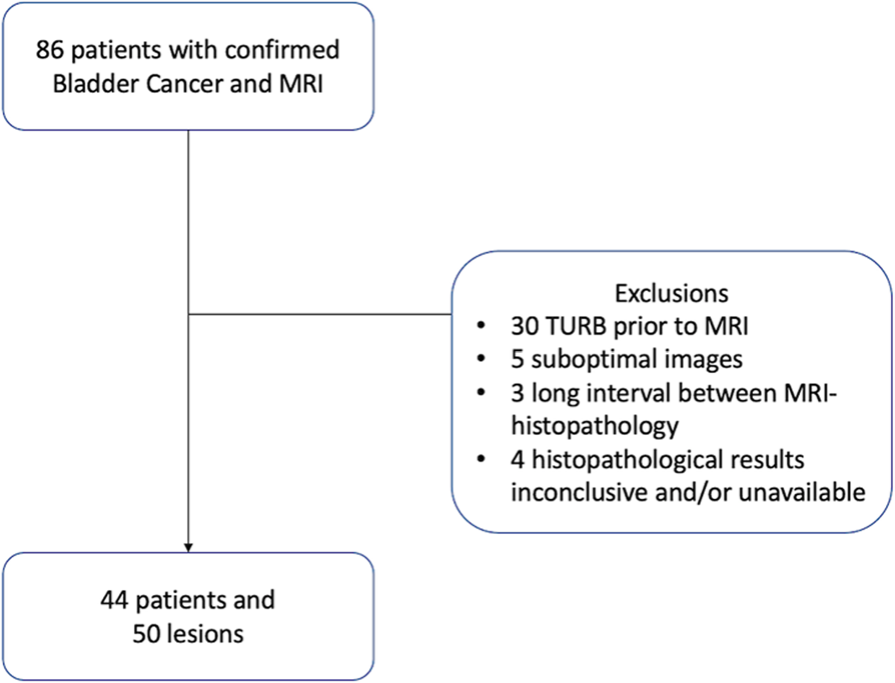
Flow chart showing the process for patients’ selection, with exclusions criteria

The search initially retrieved 86 consecutive patients. The exclusion criteria were a) patients with TURBT prior to the MRI (=30); b) suboptimal images for analysis (=5), c) an interval between MRI and surgery/TURBT longer than 4 moths (=3), and d) histopathological results that were inconclusive or unavailable (=4). After exclusions, 44 patients with 50 lesions were enrolled in the study. A flow chart is shown in Fig. 1.

### MRI protocol

All the exams were performed with a 16-channel, 1.5-T MRI scanner (Philips Achieva, Best - The Netherlands) using a 16-channel body coil placed around the pelvis. The MRI protocol was set according to the VI-RADS protocol [12]. High-resolution T2-weighted images were obtained in the axial, sagittal, and coronal planes, with 3.0 mm slice thickness; DWI images were obtained in the axial and sagittal planes using four b-values: 0,250,500, and 1000 s/mm^2^. Volumetric T1 images were obtained in the axial plane. The images were acquired before intravenous contrast injection and at 30, 60, and 120 s after. In 3 cases, the DCE sequences were not performed due to patients’ contraindications.

### MRI analysis

Two readers independently reviewed images from all patients. Both were radiologists: one was in the first year after completion of a fellowship in abdominal imaging, and the second had 21 years of experience in abdominal imaging. Both were blinded to clinical and histopathological data but were aware about the indication of MRI (i.e., staging BCa). The analysis was divided into two sessions. On the first one, all the images except the DCE sequences were available. The second session was held 3 months later and included the full protocol, with DCE images. The analysis encompassed a subjective evaluation of the anatomy of the lesions. Concerning the form, the lesions were classified as papillary (either broad base or pedunculated) or flat lesions. For the papillary lesions, readers noted the ones that showed a stalk.

Subsequently, both readers classified lesions according to the VI-RADS score for T2, DWI, and DCE images and also for the final VI-RADS category [12]. The VI-RADS score was interpreted as follows: category 1: muscle invasion is highly unlikely; category 2: muscle invasion is unlikely; category 3: muscle invasion is indeterminate; category 4: muscle invasion is likely; and category 5: muscle invasion is very likely.

### Clinical data

A third radiologist collected all clinical data from all patients by accessing HIS system. The reader was a fellow in abdominal imaging. The data collected included the date of MRI and surgery or TURB with the subsequent interval.

### Histopathological analysis

All surgical procedures, TURBT, and radical cystectomy were performed by the same group of urologists from our institution, and led by a urologist devoted to oncologic surgeries with more than 20 years of experience. The standard of reference for this study was histopathological studies derived from surgery, radical cystectomy (*n* = 26, 59.1%), and TURB (*n* = 18, 40.9%). The pathological specimens were reviewed for this study by an experienced pathologist, with 18 years of experience. For definition of muscular invasion, the criteria proposed by the American Joint Committee on Cancer [21].

### Statistical analysis

Statistical analysis was carried out using the software Stata version 15.0. The categorical variables were expressed as proportions, and comparisons were performed using the chi-squared test. Quantitative variables were presented with the mean and standard deviation if normally distributed. Comparisons between continuous variables were performed using the student’s t-test or Mann-Whitney test.

For the assessment of diagnostic accuracy, we combined stages T2, T3, and T4 into one group (MIBCa), and T1 lesions composed the other group (NMIBCa). After that, the diagnostic accuracy was estimated using a 2 × 2 contingency table. We also compared the diagnostic accuracy of both readers and the whole group of lesions with the accuracy from small lesions of up to 3.0 cm [4, 22] since these lesions are more challenging when staging.

For the reproducibility evaluation, ICC was used to assess the agreement between observers for the presence of a stalk; the VI-RADS score in T2, DWI, and DCE; and the final VI-RADS. The obtained reliability was interpreted as follows: 0.01–0.50, none to poor reliability; 0.50–0.75, moderate reliability; 0.75–0.90, good reliability; and 0.91–1.0, excellent reliability [23]. For all comparisons, a significance level of 5% (*p* < 0.05) was adopted.

## Results

**Fig. 2:**
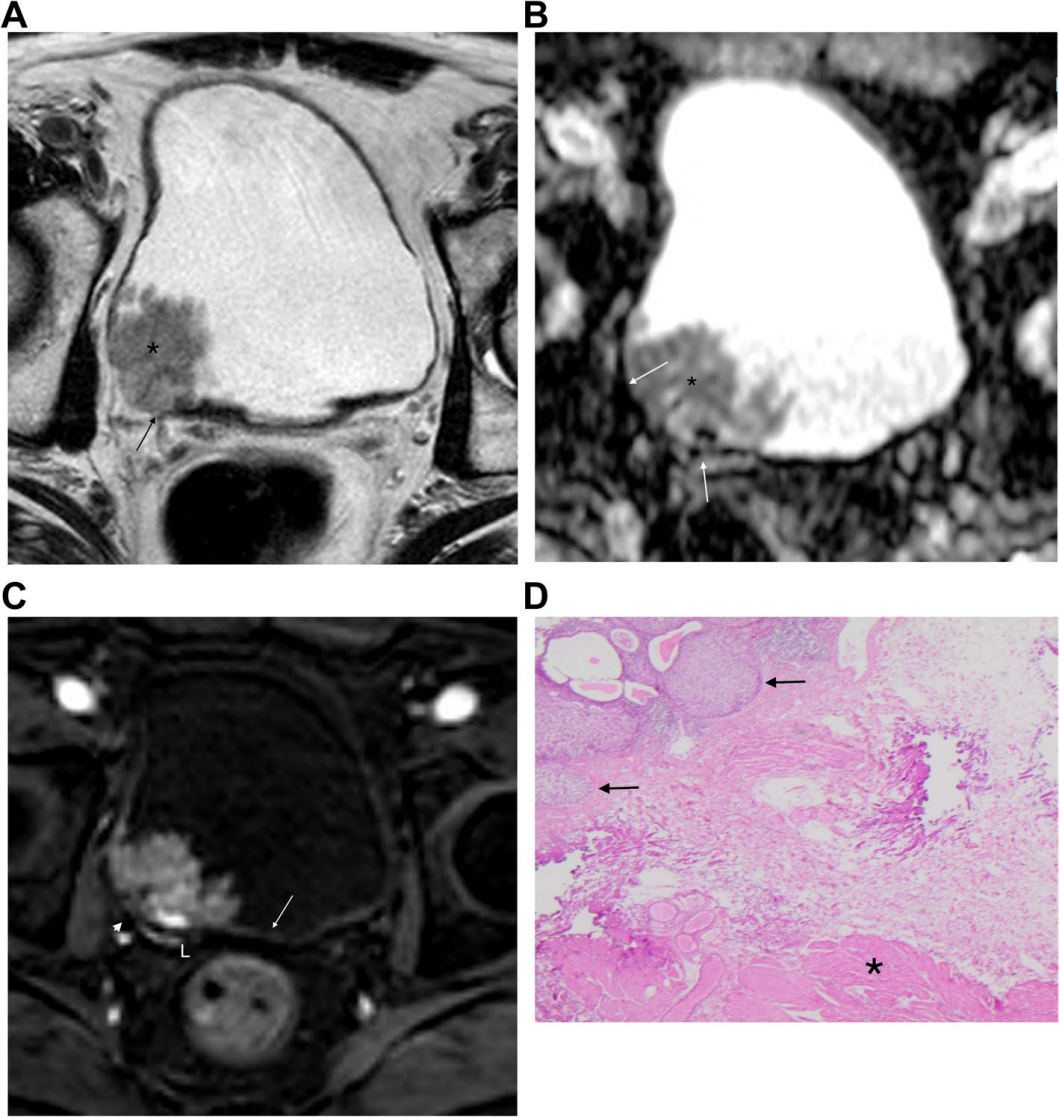
**a-d** A 78 y/o male, with a polypoid lesion in posterolateral face of bladder, diagnosed as a urothelial carcinoma. **a.** T2-weigthed image in axial plane showing the lesion (*) protruding to bladder lumen. Apparently, there is discontinuity of low signal of muscle layer (arrow) suggesting invasion. In **b**, in this ADC map, it is possible to see the low signal of muscle layer (white arrows) and in **c** in DCE, arterial phase, there is enhancement of the inner layer (white arrow), without involvement of muscle layer, which is seen low signal, at this time (arrowhead); **d.** Hematoxilin- Eosin (H&E, 100x). The urothelial lesion (white arrows) is seen away from the muscular propria (star) of the bladder, which is clearly spared. A high-grade urothelial lesion, without muscle involvement, was confirmed after TURBT

**Fig. 3:**
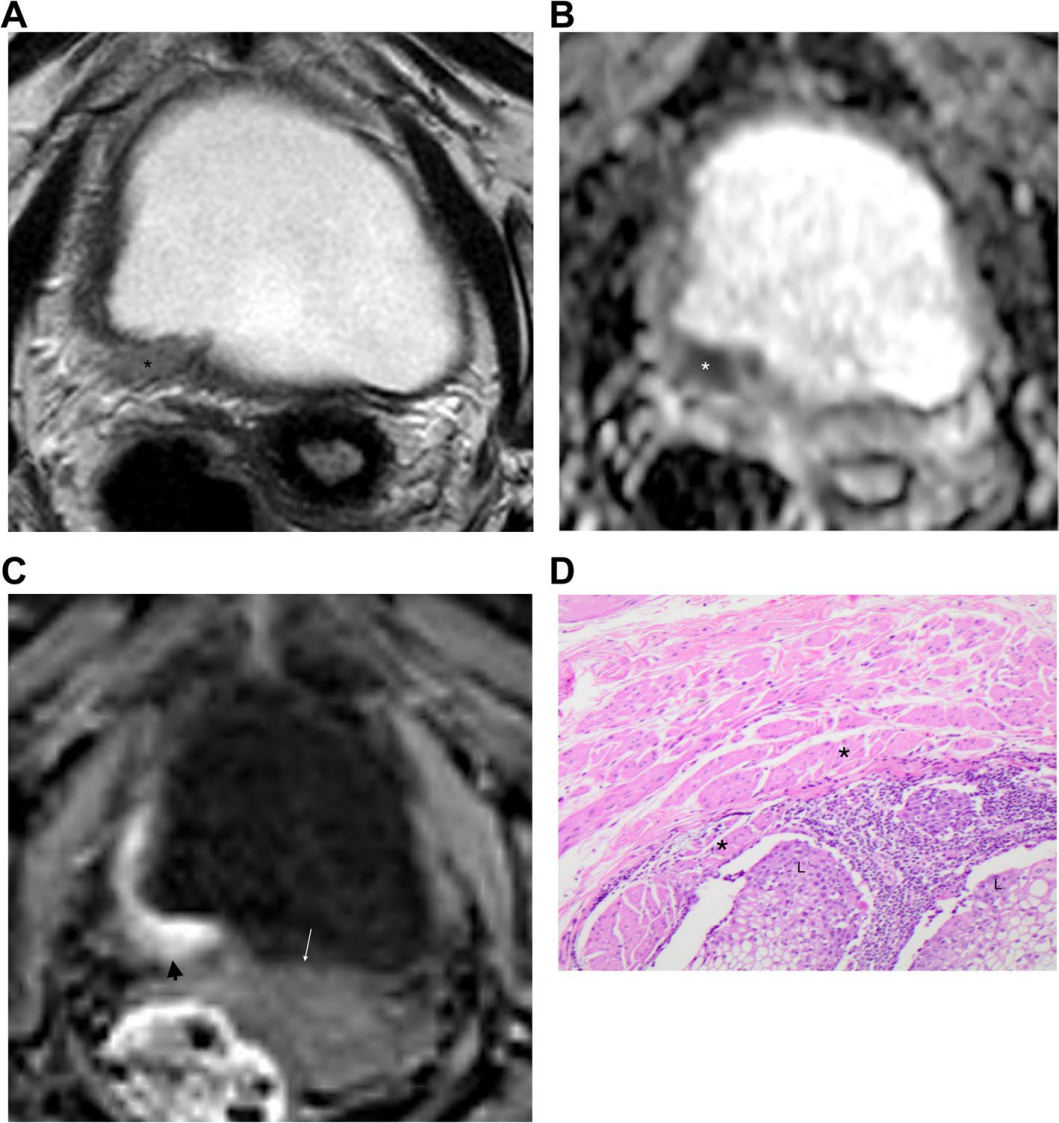
**a-d**- A 65 y/o female, presented with macroscopic hematuria. **c.** T2-weigthed image in axial plane showing a flat lesion (*) in the right posterior bladder wall, protruding through the muscle layer. **b.** In ADC map, the lesion is seen extending across the whole wall, with interruption of low signal of *muscularis propria*; **c.** Arterial phase, after intravenous contrast media injection, showing enhancement of the lesion (arrowhead), underneath the lamina propria (white arrow), confirming the muscular involvement. **d.** In this Hematoxilin- Eosin (H&E, 100x), the lesion (L) is infiltrating the *muscularis propria* of the bladder (asterisk). A high-grade urothelial cancer invading the muscular (T2 staging) was confirmed after cystectomy

Of the 44 patients, 25 were female (56.8%) and 19 were male (43.2%). The mean age was 68.7 +/− 10.8 years, ranging from 35 to 91 years. The pathologic staging was distributed as follows: 25 cases (50.0%) were T1 (Fig. 2); 8 cases (16.0%) were T2; 8 cases (16.0%) were T3; and 9 cases (18.0%) were T4. Accordingly, 25 lesions (50%) were NMIBCa, while the other half had muscular involvement (Fig. 3). The demographic data for both groups are presented in Table 1. The standard of reference was surgery and pathologic specimens in 26 patients (59.1%) and histopathological analysis from TURB in 18 patients (40.9%). The final diagnosis was high-grade urothelial cancers for 43 lesions (86.0%), low-grade urothelial cancer for 5 lesions (10.0%), and other histologic subtypes for 2 lesions (4.0%).

**Table 1:** Demographic data according to group with and without muscle invasion

	NMIBCa ***n*** = 20	MIBCa ***n*** = 24	***p***-value
**Age (years)**	72.9 +/− 8.72 (61–91)	65.2 +/− 11.3 (35–81)	*p* = 0.01
**Gender**	female = 11 (55.0%) male = 9 (45.0%)	female = 14 (58.3%) male = 10 (41.7%)	*p* = 0.82
**Interval MRI/pathology (days)**	41.4 +/− 39.1 (1–118) median = 41	35.1 +/− 35.2 (1–116) median = 35	*p* = 0.51

*NMIBCa* Non-muscle invasive bladder cancer, *MIBCa* Muscle-invasive bladder cancer, *MRI* Magnetic Resonance Imaging

The MRI findings are presented in Table 2. There was no significant difference between the two groups in the presence of a stalk for both readers (*p* = 0.16 for both reader). The form of the lesion was significantly different for both readers. For both readers, in the NMIBCa group, 23 lesions (92.0%) were papillary and only 2 (8.0%) were flat. In the MIBCa group, for reader 1, 16 (64.0%) were papillary and 9 were flat (36.0%) (*p* = 0.01), and for reader 2, 17 (68.0%) were papillary and 8 were flat (32.0%), and the difference was also significant (*p* = 0.03). For reader 1, the mean size in NMIBCa was 2.42 +/− 1.58 cm, and for the MIBCa group, it was 5.70 +/− 2.67 cm (*p* < 0.0001). For reader 2, the size was 2.37 +/− 1.50 cm vs. 5.44 +/− 2.90 cm, respectively (*p* < 0.0001).

**Table 2:** MRI parameters according to groups with and without muscle invasion, for both readers (reader 1 top line; reader 2 bottom line)

	NMIBCa ***n*** = 25	MIBCa ***n*** = 25	***p***-value
**Presence of stalk**	4 (16.0%)	1 (4.0%)	*p* = 0.16
	4 (16.0%)	1 (4.0%)	*p* = 0.16
**Form**	papillary - 23 (92.0%) flat - 2 (8.0%)	papillary - 16 (64.0%) flat - 9 (36.0%)	*p* = 0.01
	papillary - 23 (92.0%) flat - 2 (8.0%)	papillary - 17 (68.0%) flat - 9 (32.0%)	*p* = 0.03
**Size (cm)**	2.42 +/− 1.58 (0.7–7.0)	5.70+/− 2.67 (2.2–14.0)	*p* < 0.0001
	2.37 +/− 1.50 (0.7–6.8)	5.44 +/− 2.90 (1.6–14.8)	*p* < 0.0001

*NMIBCa* Non-muscle invasive bladder cancer, *MIBCa* Muscle-invasive bladder cancer, *MRI* Magnetic Resonance Imaging

The Table 3 shows the distribution of mpVI-RADS score according to pathological status, and the risk of muscle invasion for VI-RADS 2,3,4 and 5 was 7.1, 0, 55.5 and 86.4%, respectively for reader 1 and 6.3, 25.0, 63.6 and 94.1% for reader 2.

**Table 3:** Distribution of cases by each VI-RADS final category and correlation with final histopathological definition of muscular layer status

	Reader 1		Reader 2	
	Muscular Invasion			
	–	+	–	+
**VI-RADS 1**	2	0	2	0
**VI-RADS 2**	13	1	15	1
**VI-RADS 3**	3	0	3	1
**VI-RADS 4**	4	5	4	7
**VI-RADS 5**	3	19	1	16
**Total**	25	25	25	25

(−) - absent, (+) - present

Table 4 shows the diagnostic accuracy of the final VI-RADS classification for both readers, for all lesions and the small lesions only. A trend was observed towards a better performance of the more experienced reader for final mpVI-RADS. The area under the curve (AUC) for muscle invasion with mpVI-RADS, considering all lesions, was similar for both readers, for reader 1 was 0.885 +/− 0.04 (95% CI-0.79-0.98) for reader 1 and 0.924 +/− 0.04 (0.84–0.99) for reader 2, and for bpVI-RADS was 0.879+/− 0.05 and 0.916 +/− 0.04 (0.85–0.99), respectively, both differences not statistically significant (*p* = 0.24 and 0.07, respectively). When subgroup analysis was performed (Table 4), considering only lesions up to 3.0 cm, a drop in the overall accuracy of mpVI-RADS was observed for both readers, the accuracy for mpVI-RADS was 0.795 +/− 0.11 (0.57–1.0) for reader1, and 0.80 +/− 0.11(0.57–1.0) for reader 2, a non-significant difference (*p* = 0.56). However, for bpVI-RADS the AUC was 0.747 +/− 0.12 (0.50–0.99) for reader 1 and 0.80 +/− 0.11(0.57–1.0) for reader 2, a significant difference (*p* = 0.04.

**Table 4:** Diagnostic accuracy assessed by area under the curve (AUC) for the whole sample and considering only small lesions (< 3.0 cm)

	Reader 1		Reader 2		***p***-value R1 x R2 Small Lesions	***p***-value R1 x R2 All Lesions
	Small lesions (***n*** = 25)	All cases (***n*** -50)	Small lesions (***n*** = 25)	All cases (***n*** = 50)		
**bpVI-RADS**	0.747 +/− 0.12 (0.50–0.99)	0.879+/−0.05 (0.78–0.95)	0.800 +/− 0.11 (0.57–1.0)	0.916 +/− 0.04 (0.85–0.99)	0.04	0.07
**mpVI-RADS**	0.795 +/− 0.11 (0.57–1.0)	0.885 +/− 0.04 (0.79–0.98)	0.800 +/− 0.11 (0.57–1.0)	0.924 +/− 0.04 (0.84–0.99)	0.56	0.24
**Intra-reader** *p*-value*	0.23	0.57	0.18	0.24		

*bpVI-RADS* - biparametric VI-RADS - Analysis without post-contrast sequences

mpVI-RADS - multiparametric VI-RADS - Analysis with post-contrast sequences

The inter-reader agreement for the presence of a stalk in the lesions was 0.77 (95%CI: 0.51–1.0), while for the lesions’ form was 0.94 (0.68–1.0), and the for the final mpVI-RADS score was 0.81 (0.60–1.0). For the final score using the biparametric approach the kappa value was 0.85 (0.63–1.0). When assessing agreement in each sequence, the best result was obtained for DCE with an ICC of 0.85 (0.59–1.0), indicating excellent agreement. These values and those of ICC for the small lesions group are showed in Table 5.

**Table 5:** - Intraclass Correlation Coefficient (ICC) for the imaging parameters and for the VI-RADS sequences and final score

Parameter	All lesions		Small lesions	
	ICC (95% CI)	***p***-value	ICC (95% CI)	***p***-value
**Stalk**	0.77 (0.51–1.00)	*p* < 0.0001	1.00=/− 0.20	*p* < 0.0001
**Form**	0.94 (0.68–1.00)	*p* < 0.0001	0.87 +/− 0.18	*p* < 0.0001
**VI-RADS T2**	0.83 (0.61–0.85)	*p* < 0.0001	0.79+/−0.14	*p* < 0.0001
**VI-RADS DWI**	0.78 (0.59–0.99)	*p* < 0.0001	0.74+/−0.14	*p* < 0.0001
**VI-RADS DCE**	0.85 (0.59–1.00)	*p* < 0.0001	0.85+/−0.14	*p* < 0.0001
**mpVI-RAD Final**	0.81 (0.60–1.00)	*p* < 0.0001	0.82 (0.71–0.95)	*p* < 0.0001
**bpVI-RADS Final**	0.85 (0.63–1.00)	*p* < 0.0001	0.85 (0.70–0.95)	*p* < 0.0001

*DWI* diffusion-weighted imaging, *DCE* dynamic contrast-enhanced, *CI* Confidence Interval

## Discussion

Our results suggest that the use of intravenous contrast media only improves the diagnostic accuracy of bladder cancer staging, using the VI-RADS system score, when less experienced readers assess small lesions.

In our study, we assumed that a VI-RADS score of only 4 indicates muscle-invasive disease. There were two reasons for this. First, the statistical analysis pointed to this threshold as the optimal point for better accuracy when determining MIBCa. Second, the decision to perform bladder resection should be based on a more specific approach.

Interestingly, despite the low number of cases in each category in our cohort, the false-negative rates were only 4.0 and 8.0% (cases classified as VI-RADS 1, 2 and 3) for the less and more experienced readers, respectively. However, the rates of false-positive cases were relatively high 28.0 and 20.0% for readers 1 and 2, respectively. Our results are aligned with previous studies [15, 17, 22], but slightly better performance was obtained for false-negative cases, although slightly worse results were obtained for false-positive cases [17, 23].

When comparing the accuracy of readers, the results of the less experienced one were consistently inferior to those of the more experienced reader throughout the different sequences and for the final score, but the difference was significant only when assessing small lesions with biparametric approach. One possible explanation for these results is that our study may be underpowered for showing significant difference in other scenarios. Or, biparametric approach may have similar accuracy for experienced readers, which would be not surprising as, in many situations, the diagnostic accuracy of an imaging method is clearly dependent on the experience of the reader [23]. Highly-specialized exams (for instance, prostate MRI) have a known learning curve for interpretation, and some organizations have suggested minimum requirements for independent reporting. However, this is an open issue for bladder cancer imaging [24].

We decided to perform a subgroup analysis assessing the diagnostic accuracy for small bladder lesions (≤ 3.0 cm) because the staging tends to be more complex in these cases. The difference in diagnostic accuracy of this limited sample was not significant, but compared to the whole group, there was a clear trend of lower accuracy for both reader 1 (84.0 vs. 79.2) and reader 2 (86.0 vs. 79.2) for the final mpVI-RADS score and for the bpVI-RADS, 79.6 vs. 75.0 (reader 1) and 85.7 vs. 79.2 (reader 2). This trend was even more conspicuous, although without statistical significance for the less experienced reader, which may indicate, depending on further studies that multiparametric approach should be preserved when radiologists with limited experience were scanning and interpreting MR studies, mainly if small lesions were detected on anatomical sequences. Once the situations where the use of contrast-enhanced sequences are clear, a more rational approach (including these two protocols) for bladder cancer staging would be feasible.

In the pre-VI-RADS years, some relevant studies have placed the contrast-enhanced sequences as essential for the best results on vesical cancer staging [10, 11]. However, recently, the role of contrast sequences has been questioned. Delle Pizzi et al. [19] in a multi-reader, a prospective study found similar accuracies for MRI protocol with and without contrast media, regardless of readers’ experience. Gmeiner et al. [20] pointed out the same direction, although in a retrospective study and using a different protocol than suggested in VI-RADS [12]. Our study also indicates the same direction. Possible explanations for this change in the perception of the value of contrast sequences include the improvement in MRI techniques, including the non-Gradient Echo DWI sequences, the motion-corrected high-resolution T2-weighted images, and the ascending learning curve of all radiologists as Bladder cancer staging is a much more requested exam now than a decade ago.

The VI-RADS scoring system was designed with a focus on the standardization of MR imaging for staging BCa. Based on a multisequence approach, the system also defines an algorithm to conduct cases using findings from different image acquisitions in a hierarchical approach [12]. By using findings from different sequences, one would expect a quite variable interpretation, but the literature has shown excellent reproducibility for the final assessment of the muscle layer by VI-RADS [17, 25, 26] ranging from 0.73 to 0.92. Our data are aligned with the literature in showing substantial agreement for final classification with an ICC of 0.81 (0.60–1.00), *p* < 0.0001, for all lesions and 0.82 (0.71–0.95), *p* < 0.0001 for lesions under 3.0 cm. On the other side, in our study, in contrast to others in literature [17, 25, 27], DCE showed almost perfect agreement, slightly higher than those observed for T2, DWI and the final VI-RADS score. This represents an interesting point, as DCE and DWI are the sequences that define the final scores when discrepant findings are present, according to the algorithm proposed by VI-RADS. And, based on it, we could argue that DCE can be preserved in specific situations (small lesions/less experienced readers) as a problem-solving sequence with a high level of reproducibility.

Our study does have some limitations that should be acknowledged. First, it is a retrospective study with all inherent risks of bias, although we carefully applied inclusion and exclusion criteria to minimize them. Second, we had a small cohort, which may limit the analysis of subgroups, such as some of the five categories of VI-RADS. The reason for the small number of patients in our sample was the high number of patients who had cystoscopy/TURB prior to MRI in the first year of our study, which reflects that urologist were not so familiar with VI-RADS at that time. Third, we performed all examinations with a 1.5-T scanner. However, the exclusive use of 3.0-T scanners has not been recommended by VI-RADS, and a recent meta-analysis showed comparable results between studies performed using 1.5 T and 3.0 T [28].

## Conclusion

Our results indicate good overall accuracy for distinguishing NMIBCa from MIBCa using the biparametric approach, similar to the mpVI-RADS original score. However, a trend towards a lower overall accuracy with a biparametric approach, more evident for small lesions and for the less experienced reader, may indicate that DCE sequences should be preserved in these situations. Further, larger, prospective, and multi-institutional studies are required for defining the exact role of contrast sequences in Bladder cancer staging.

## Data Availability

The datasets during and/or analyzed during the current study available from the corresponding author on reasonable request.

## Abbreviations

AUC: Area under the curve
BCa: Bladder Cancer
Bp: biparametric
CT: Computed Tomography
DCE: Dynamic contrast-enhancement
DWI: Diffusion-weighted imaging
ICC: Intra-class coefficient
HIS: Hospital Information System
Mp: Multiparametric
MIBC: Muscle-invasive bladder cancer
MRI: Magnetic Resonance Imaging
NMIBC: Non-muscle-invasive bladder cancer
RIS: Radiological Information System
TURBT: Transurethral resection of bladder tumors
VI-RADS: Vesical Imaging, Reporting and Data System
